# Supplementary figures and images for: Polycaprolactone nanofibers as an adjuvant strategy for Tamoxifen release and their cytotoxicity on breast cancer cells
Source: PeerJ. 2021 Oct 27;9:e12124. doi: 10.7717/peerj.12124 (PMC8556714; doi:10.7717/peerj.12124)

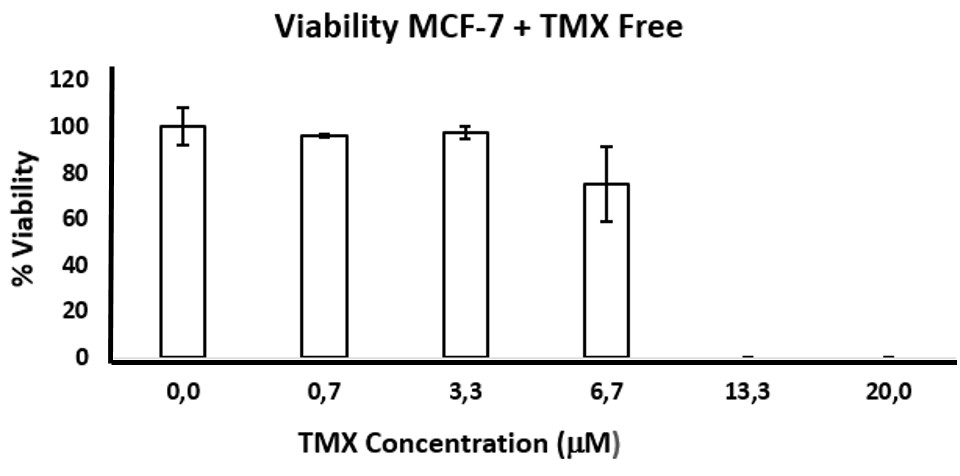

Supplement: Supplemental Information 3 — Cytotoxic effect on MCF-7 cells was determined after 1 day of exposure to TMX (0–20 µM). The reduction of resazurin to resorufin was measured at 4 h. [file peerj-09-12124-s003.jpg]
